# Supplementary material for: Whole-Exome Sequencing Reveals Migraine-Associated Novel Functional Variants in Arab Ancestry Females: A Pilot Study
Source: Brain Sci. 2022 Oct 24;12(11):1429. doi: 10.3390/brainsci12111429 (PMC9688800; doi:10.3390/brainsci12111429)
Supplement: Supplementary file 1 [file brainsci-12-01429-s001.zip › brainsci-1954467-supplementary.pdf]

Whole-Exome Sequencing Reveals Migraine-Associated Novel Functional Variants in Arab Ancestry Females: A Pilot Study

Johra Khan [1,2,*], Lubna Al Asoom [3], Ahmad Al Sunni [3], Nazish Rafique [3], Rabia Latif [3], Majed Alabdali [4], Azhar Alhariri [5], Majed Aloqaily [5], Sayed AbdulAzeez [5], Sadaf Jahan [1], Saeed Banawas [1,2,6] and J. Francis Borgio [5,7]

[1] Department of Medical Laboratory Sciences, College of Applied Medical Sciences, Majmaah University, Majmaah 11952, Saudi Arabia
[2] Health and Basic Sciences Research Center, Majmaah University, Majmaah 11952, Saudi Arabia
[3] Department of Physiology, College of Medicine, Imam Abdulrahman Bin Faisal University, Dammam 31541, Saudi Arabia
[4] Department of Neurology, College of Medicine, Imam Abdulrahman Bin Faisal University, Dammam 31952, Saudi Arabia
[5] Department of Genetic Research, Institute for Research and Medical Consultations (IRMC), Imam Abdulrahman Bin Faisal University, Dammam 31441, Saudi Arabia
[6] Department of Biomedical Sciences, Oregon State University, Corvallis, OR 97331, USA
[7] Department of Epidemic Diseases Research, Institute for Research and Medical Consultations (IRMC), Imam Abdulrahman Bin Faisal University, Dammam 31441, Saudi Arabia
* Correspondence: j.khan@mu.edu.sa; Tel.: +966-538-077360

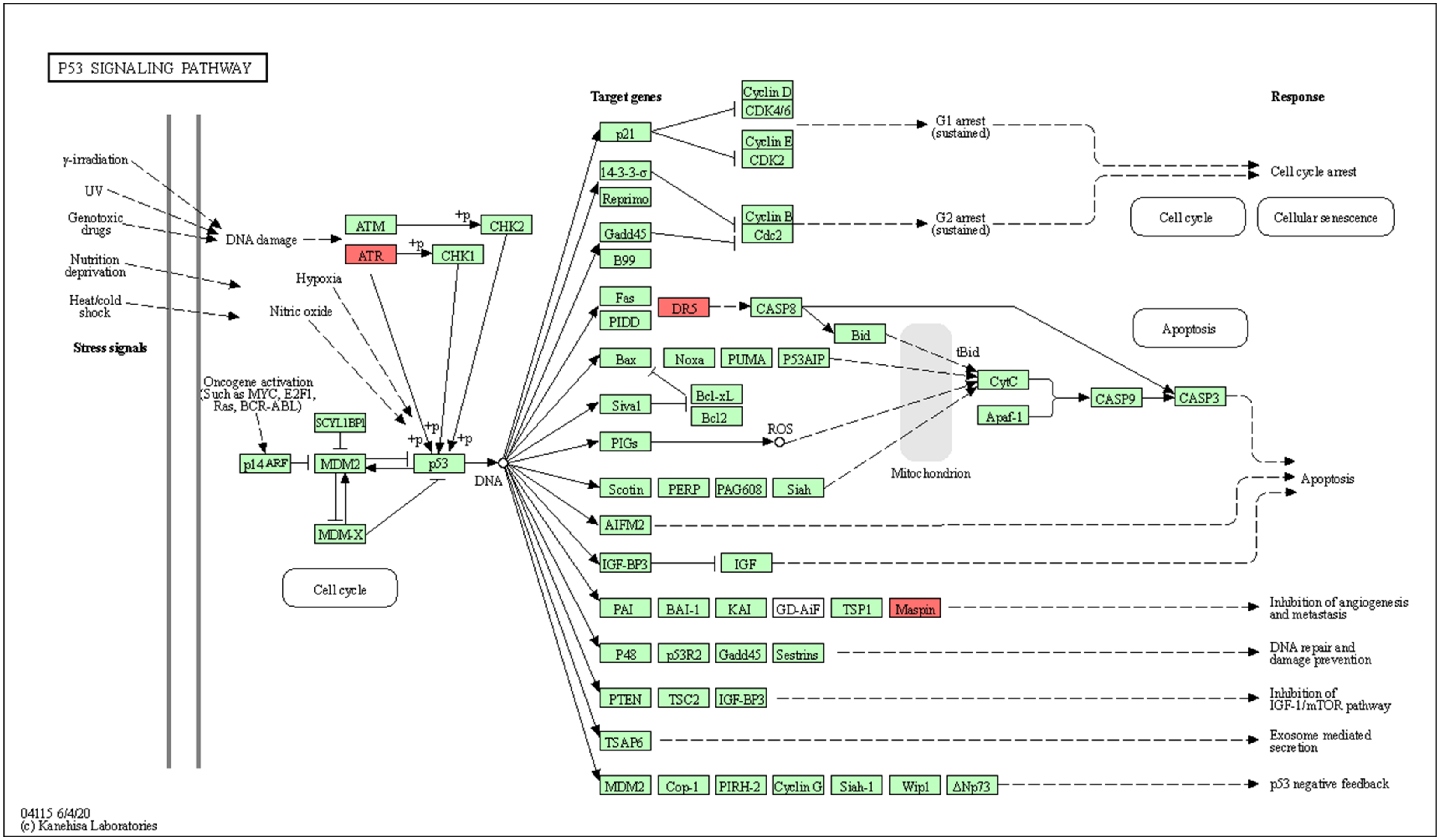

Figure S1: The p53 signaling pathway with the significant genes from the study.

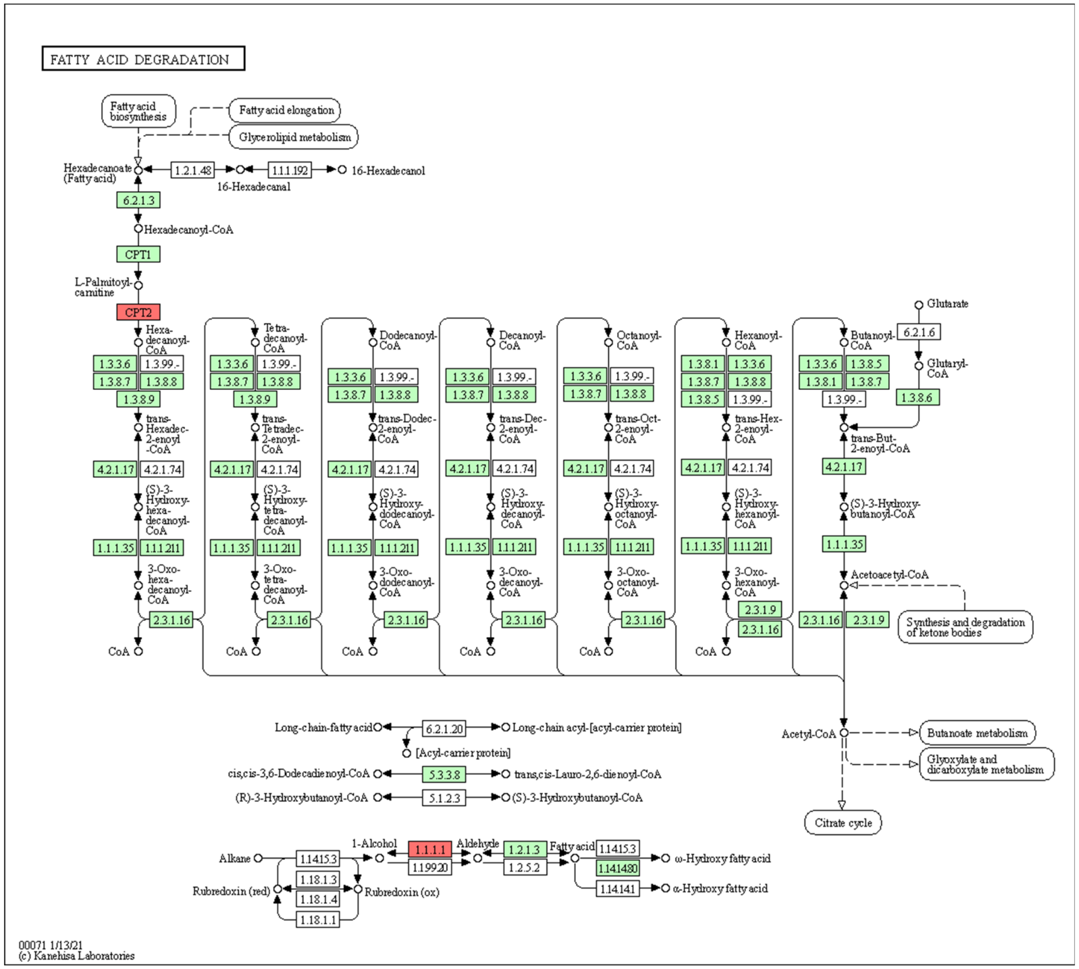

Figure S2: The Fatty acid degradation with the significant genes is the study.

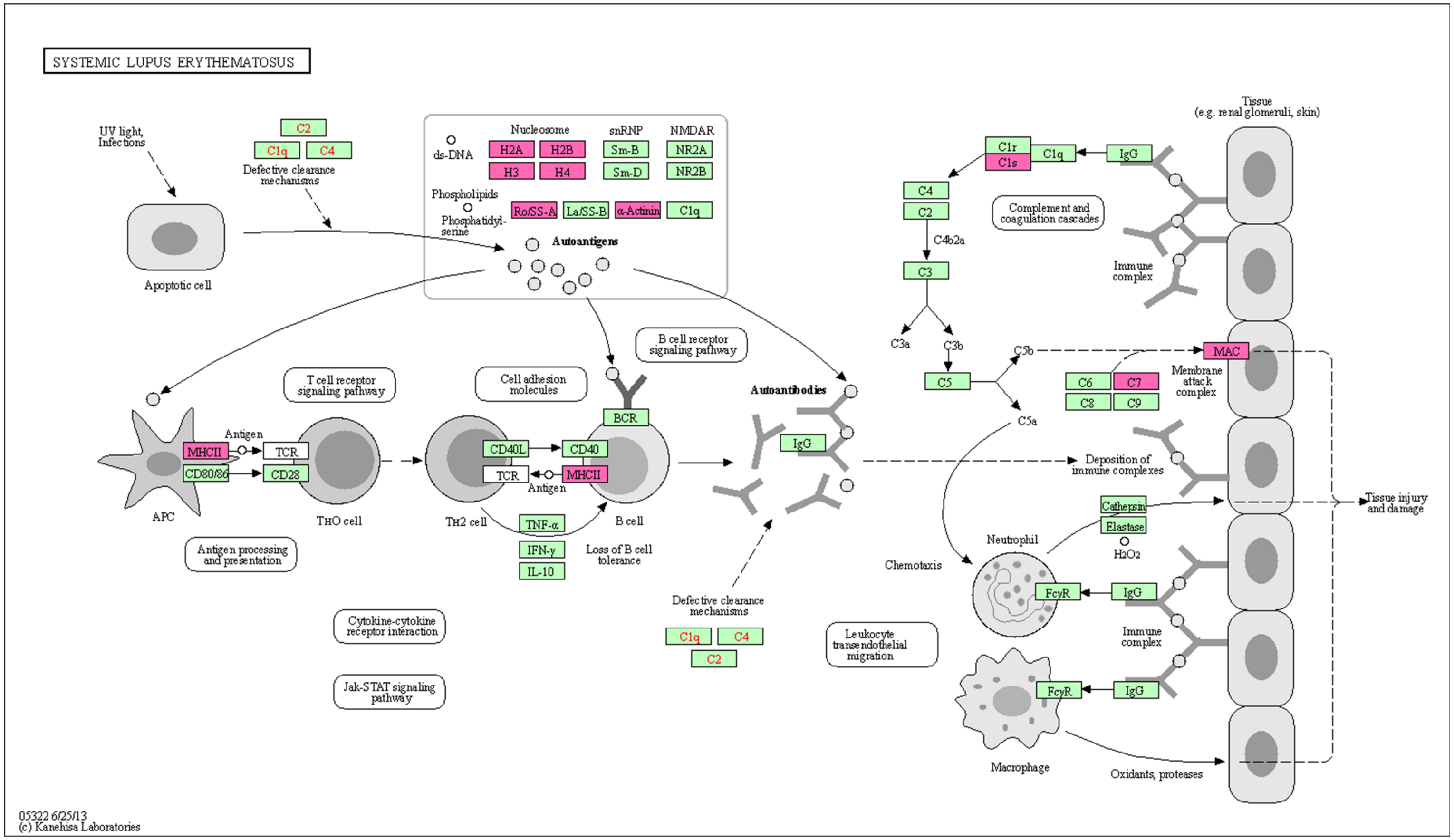

Figure S3: Systemic lupus erythematosus (hsa05322) pathway and significant genes (highlighted) identified from the study.

Supplementary Table S1: List of migraine associated (*p* value <0.00001) variants identified through exome sequencing.

SNP	locus contig	locus position	alleles	Consequence	SYMBOL	Amino acids	Protein position	Codons	*p*-value
rs772174	chr2	96325754	['A', 'G']	5_prime_UTR_variant	ITPRIPL1	-	-	-	5.2E-07
rs9878093	chr3	108755677	['A', 'C']	3_prime_UTR_variant	RETNLB	-	-	-	3.45E-06
rs5851607	chr3	108757146	['G', 'GGGGG ATTA']	stop_gained,frameshift_variant	RETNLB	-/*SX	13-14	-/TAAT CCCC	3.45E-06
rs3214023	chr12	53289202	['C', 'T']	non_coding_transcript_exon_variant	ESPL1	-	-	-	3.56E-06
rs2230354	chr17	14191992	['A', 'G']	synonymous_variant	COX10	P	233	ccA/ccG	5.32E-06
rs1455774	chr15	92472091	['T', 'G']	non_coding_transcript_exon_variant	C15orf32	-	-	-	7.96E-06
rs589292	chr9	125055997	['C', 'T']	missense_variant	SCAI	A/T	37	Gct/Act	1.17E-05
rs1126671	chr4	99127263	['T', 'C']	missense_variant	ADH4	I/V	309	Att/Gtt	1.58E-05
rs1126673	chr4	99124465	['C', 'T']	missense_variant,splice_region_variant	ADH4	V/I	393	Gtc/Atc	1.58E-05
rs1126670	chr4	99131582	['C', 'A']	synonymous_variant	ADH4	P	255	ccG/ccT	1.58E-05
rs7644369	chr3	134951682	['C', 'T']	synonymous_variant	EPHB1	S	145	agC/agT	1.67E-05
rs12318072	chr12	124314255	['C', 'T']	synonymous_variant	RFLNA	P	127	ccC/ccT	2.19E-05
rs6580942	chr12	53268840	['C', 'A']	5_prime_UTR_variant	ESPL1	-	-	-	3.41E-05
rs2229917	chr9	128218658	['G', 'A']	synonymous_variant	DNM1	E	104	gaG/gaA	3.42E-05
rs1799821	chr1	53210776	['G', 'A']	missense_variant	CPT2	V/I	368	Gtc/Atc	3.58E-05
rs3126075	chr1	152304150	['G', 'C']	missense_variant	FLG	T/R	3579	aCg/aGg	4.27E-05
rs329003	chr18	9570322	['T', 'C']	missense_variant	PPP4R1	I/V	381	Ata/Gta	4.5E-05
rs2289520	chr18	63493087	['G', 'C']	missense_variant	SERPINB5	V/L	16	Gtc/Ctc	4.56E-05
rs198460	chr11	61757502	['G', 'A']	non_coding_transcript_exon_variant	DKFZP434K028	-	-	-	5.23E-05
rs200899890	chr14	18999427	['A', 'G']	3_prime_UTR_variant	POTEM	-	-	-	5.29E-05
rs62027634	chr15	101744472	['A', 'G']	non_coding_transcript_exon_variant	LOC100128108	-	-	-	5.35E-05
rs913589	chr9	7174773	['G', 'A']	3_prime_UTR_variant	KDM4C	-	-	-	5.4E-05
rs12390	chrX	119470473	['T', 'C']	synonymous_variant	SLC25A5	T	233	acT/acC	5.5E-05
rs56358776	chr12	53288673	['G', 'A']	missense_variant	ESPL1	R/Q	1561	cGg/cAg	5.92E-05

rs12452761	chr17	73472128	['G', 'A']	synonymous_variant	SDK2	T	105	acC/acT	6.2E-05
-	chr1	196788867	['G', 'T']	non_coding_transcript_exon_variant	CFHR3	-	-	-	6.7E-05
rs7251612	chr19	20776362	['C', 'G']	5_prime_UTR_variant	ZNF66	-	-	-	7.33E-05
rs7251614	chr19	20776371	['C', 'G']	5_prime_UTR_variant	ZNF66	-	-	-	7.33E-05
-	chr19	20807177	['G', 'GACAT AAGA GAATT CATA CTGG AGAG AAAC CCTA CAAA TGTG AAGA ATGT GGCA AAGC TTTT AATC ATCC CGCA ACCC TTTT TTC']	inframe_insertion	ZNF66	R/RHK RIHT GEKP YKCE ECGK AFNH PATL FS	526	aga/ag ACAT AAGA GAAT TCAT ACTG GAGA GAAA CCCT ACAA ATGT GAAG AATG TGGC AAAG CTTT TAAT CATC CCGC AACC CTTT TTTCa	7.33E-05
rs10413187	chr19	20793848	['C', 'A']	missense_variant	ZNF66	Q/K	66	Cag/Aag	7.33E-05
rs432839	chr19	20806118	['G', 'T']	missense_variant	ZNF66	C/F	173	tGc/tTc	7.33E-05
rs383038	chr19	20806162	['T', 'C']	missense_variant	ZNF66	F/L	188	Ttt/Ctt	7.33E-05
rs370551	chr19	20806858	['A', 'G']	missense_variant	ZNF66	T/A	420	Act/Gct	7.33E-05
rs366949	chr19	20805906	['G', 'A']	synonymous_variant	ZNF66	R	102	agG/agA	7.33E-05
rs77897802	chr17	36212117	['G', 'A']	3_prime_UTR_variant	CCL4L2	-	-	-	7.77E-05

rs1270983160	chr17	36211816	['C', 'T']	synonymous_variant	CCL4L2	T	39	acC/acT	7.77E-05
rs12984041	chr19	46746039	['G', 'C']	5_prime_UTR_variant	FKRP	-	-	-	8.18E-05
rs2187473	chr11	111513121	['C', 'T']	non_coding_transcript_exon_variant	LOC728196	-	-	-	8.39E-05
rs61740794	chr2	67404699	['G', 'A']	missense_variant	ETAA1	E/K	673	Gaa/Aaa	8.51E-05
rs4876458	chr8	112224816	['C', 'T']	synonymous_variant	CSMD3	A	2159	gcG/gcA	8.72E-05
rs735943	chr1	241866849	['A', 'G']	missense_variant	EXO1	H/R	354	cAt/cGt	8.98E-05
rs17343819	chr8	67484680	['T', 'C']	missense_variant,splice_region_variant	CPA6	N/S	101	aAt/aGt	9.09E-05
rs162008	chr18	26865728	['C', 'T']	5_prime_UTR_variant	AQP4	-	-	-	9.29E-05
rs16907852	chr11	10482209	['G', 'A']	synonymous_variant	AMPD3	P	32	ccG/ccA	9.53E-05
rs72297783	chr10	23014163	['A', 'AG']	3_prime_UTR_variant	ARMC3	-	-	-	9.55E-05
rs61760973	chr11	34966796	['T', 'C']	synonymous_variant	PDHX	P	251	ccT/ccC	9.55E-05
rs3213590	chr6	167915298	['G', 'C']	non_coding_transcript_exon_variant	AFDN	-	-	-	9.97E-05
rs6906754	chr6	167917136	['A', 'C']	non_coding_transcript_exon_variant	AFDN	-	-	-	9.97E-05
rs3213590	chr6	167915298	['G', 'C']	synonymous_variant	AFDN	V	636	gtG/gtC	9.97E-05
rs6906754	chr6	167917136	['A', 'C']	synonymous_variant	AFDN	P	697	ccA/ccC	9.97E-05
rs3213590	chr6	167915298	['G', 'C']	synonymous_variant	AFDN	V	787	gtG/gtC	9.97E-05
rs6906754	chr6	167917136	['A', 'C']	synonymous_variant	AFDN	P	848	ccA/ccC	9.97E-05
rs3213590	chr6	167915298	['G', 'C']	synonymous_variant	AFDN	V	762	gtG/gtC	9.97E-05
rs6906754	chr6	167917136	['A', 'C']	synonymous_variant	AFDN	P	864	ccA/ccC	9.97E-05
rs3213590	chr6	167915298	['G', 'C']	synonymous_variant	AFDN	V	811	gtG/gtC	9.97E-05
rs6906754	chr6	167917136	['A', 'C']	synonymous_variant	AFDN	P	872	ccA/ccC	9.97E-05

Supplementary Table S2: Gene ontology pathway analysis of the top 50 genes.

**GO_Biological_Process_Enrichment**					
**Term**	**P-value**	**Adjusted P-value**	**Genes from top 50 list**	**total Genes**	**Genes**
organic hydroxy compound catabolic process (GO:1901616)	**9.12893E-05**	**0.026108731**	2	6	ADH4;CYP4F12
quinone metabolic process (GO:1901661)	**0.000272123**	**0.038913602**	2	10	ADH4;AKR1C1
regulation of insulin-like growth factor receptor signaling pathway (GO:0043567)	**0.000813271**	0.077531867	2	17	IGFBP1;CDH3
renal water homeostasis (GO:0003091)	**0.002719256**	0.172746714	2	31	AQP4;CYP4F12
adherens junction organization (GO:0034332)	**0.005418766**	0.172746714	2	44	AFDN;CDH3
diterpenoid metabolic process (GO:0016101)	**0.010526589**	0.172746714	2	62	ADH4;AKR1C1
cell-cell junction organization (GO:0045216)	**0.014004804**	0.172746714	2	72	AFDN;CDH3
regulation of cell cycle checkpoint (GO:1901976)	**0.014908285**	0.172746714	1	6	ETAA1
establishment of endothelial intestinal barrier (GO:0090557)	**0.014908285**	0.172746714	1	6	AFDN
negative regulation of transforming growth factor beta production (GO:0071635)	**0.014908285**	0.172746714	1	6	CDH3
regulation of transforming growth factor beta2 production (GO:0032909)	**0.014908285**	0.172746714	1	6	CDH3
renal system process involved in regulation of blood volume (GO:0001977)	**0.014908285**	0.172746714	1	6	CYP4F12
retinoid metabolic process (GO:0001523)	**0.015132736**	0.172746714	2	75	ADH4;AKR1C1
positive regulation of insulin-like growth factor receptor signaling pathway (GO:0043568)	**0.017371741**	0.172746714	1	7	CDH3
positive regulation of keratinocyte proliferation (GO:0010838)	**0.017371741**	0.172746714	1	7	CDH3
positive regulation of mitotic sister chromatid separation (GO:1901970)	**0.017371741**	0.172746714	1	7	ESPL1
long-chain fatty acid catabolic process (GO:0042758)	**0.017371741**	0.172746714	1	7	CYP4F12
granulocyte migration (GO:0097530)	**0.017371741**	0.172746714	1	7	ADGRE2
positive regulation of metaphase/anaphase transition of cell cycle (GO:1902101)	**0.017371741**	0.172746714	1	7	ESPL1
intestinal cholesterol absorption (GO:0030299)	**0.019829161**	0.172746714	1	8	AKR1C1
positive regulation of mitotic metaphase/anaphase transition (GO:0045842)	**0.019829161**	0.172746714	1	8	ESPL1

daunorubicin metabolic process (GO:0044597)	**0.019829161**	0.172746714	1	8	AKR1C1
aminoglycoside antibiotic metabolic process (GO:0030647)	**0.019829161**	0.172746714	1	8	AKR1C1
doxorubicin metabolic process (GO:0044598)	**0.019829161**	0.172746714	1	8	AKR1C1
histone H3-K36 demethylation (GO:0070544)	**0.019829161**	0.172746714	1	8	KDM4C
intestinal lipid absorption (GO:0098856)	**0.022280559**	0.172746714	1	9	AKR1C1
regulation of leukocyte degranulation (GO:0043300)	**0.022280559**	0.172746714	1	9	ADGRE2
regulation of mast cell activation involved in immune response (GO:0033006)	**0.022280559**	0.172746714	1	9	ADGRE2
regulation of hair cycle (GO:0042634)	**0.022280559**	0.172746714	1	9	CDH3
regulation of DNA damage checkpoint (GO:2000001)	**0.024725949**	0.172746714	1	10	ETAA1
establishment of spindle localization (GO:0051293)	**0.024725949**	0.172746714	1	10	ESPL1
formation of extrachromosomal circular DNA (GO:0001325)	**0.027165345**	0.172746714	1	11	EXO1
carnitine shuttle (GO:0006853)	**0.027165345**	0.172746714	1	11	CPT2
replicative senescence (GO:0090399)	**0.027165345**	0.172746714	1	11	ATR
protein O-linked mannosylation (GO:0035269)	**0.027165345**	0.172746714	1	11	FKRP
regulation of myeloid leukocyte mediated immunity (GO:0002886)	**0.027165345**	0.172746714	1	11	ADGRE2
fatty acid transmembrane transport (GO:1902001)	**0.027165345**	0.172746714	1	11	CPT2
retinal metabolic process (GO:0042574)	**0.027165345**	0.172746714	1	11	AKR1C1
activation of cysteine-type endopeptidase activity involved in apoptotic signaling pathway (GO:0097296)	**0.027165345**	0.172746714	1	11	TNFRSF10B
alcohol catabolic process (GO:0046164)	**0.027165345**	0.172746714	1	11	ADH4
telomere maintenance via telomere trimming (GO:0090737)	**0.027165345**	0.172746714	1	11	EXO1
t-circle formation (GO:0090656)	**0.027165345**	0.172746714	1	11	EXO1
progesterone metabolic process (GO:0042448)	**0.027165345**	0.172746714	1	11	AKR1C1
PERK-mediated unfolded protein response (GO:0036499)	**0.029598762**	0.172746714	1	12	IGFBP1
histone H3-K9 demethylation (GO:0033169)	**0.029598762**	0.172746714	1	12	KDM4C
prostanoid metabolic process (GO:0006692)	**0.029598762**	0.172746714	1	12	AKR1C1
regulation of sister chromatid cohesion (GO:0007063)	**0.029598762**	0.172746714	1	12	ESPL1
retinol metabolic process (GO:0042572)	**0.029598762**	0.172746714	1	12	ADH4
water transport (GO:0006833)	**0.032026213**	0.172746714	1	13	AQP4
fluid transport (GO:0042044)	**0.034447714**	0.172746714	1	14	AQP4
glycoside metabolic process (GO:0016137)	**0.034447714**	0.172746714	1	14	AKR1C1

positive regulation of DNA damage response, signal transduction by p53 class mediator (GO:0043517)	**0.034447714**	0.172746714	1	14	ATR
negative regulation of Rho protein signal transduction (GO:0035024)	**0.034447714**	0.172746714	1	14	SCAI
positive regulation of protein serine/threonine kinase activity (GO:0071902)	**0.035176955**	0.172746714	2	118	ETAA1;TNFRSF10B
DNA replication (GO:0006260)	**0.036270617**	0.172746714	2	120	EXO1;ATR
positive regulation of cysteine-type endopeptidase activity involved in apoptotic signaling pathway (GO:2001269)	**0.036863279**	0.172746714	1	15	TNFRSF10B
intracellular lipid transport (GO:0032365)	**0.036863279**	0.172746714	1	15	CPT2
protein localization to chromosome, telomeric region (GO:0070198)	**0.036863279**	0.172746714	1	15	ATR
regulation of mast cell degranulation (GO:0043304)	**0.036863279**	0.172746714	1	15	ADGRE2
regulation of mitotic metaphase/anaphase transition (GO:0030071)	**0.036863279**	0.172746714	1	15	ESPL1
activation of NF-kappaB-inducing kinase activity (GO:0007250)	**0.039272921**	0.172746714	1	16	TNFRSF10B
regulation of water loss via skin (GO:0033561)	**0.039272921**	0.172746714	1	16	FLG
establishment of skin barrier (GO:0061436)	**0.039272921**	0.172746714	1	16	FLG
negative regulation of DNA replication (GO:0008156)	**0.039272921**	0.172746714	1	16	ATR
positive regulation of hormone secretion (GO:0046887)	**0.041676655**	0.172746714	1	17	GRP
positive regulation of G-protein coupled receptor protein signaling pathway (GO:0045745)	**0.041676655**	0.172746714	1	17	GRP
protein mannosylation (GO:0035268)	**0.041676655**	0.172746714	1	17	FKRP
positive regulation of signal transduction by p53 class mediator (GO:1901798)	**0.041676655**	0.172746714	1	17	ATR
cellular response to gamma radiation (GO:0071480)	**0.041676655**	0.172746714	1	17	ATR
regulation of keratinocyte proliferation (GO:0010837)	**0.044074495**	0.175073688	1	18	CDH3
establishment of mitotic spindle localization (GO:0040001)	**0.044074495**	0.175073688	1	18	ESPL1
response to organophosphorus (GO:0046683)	**0.044074495**	0.175073688	1	18	AKR1C1
endoplasmic reticulum mannose trimming (GO:1904380)	**0.046466455**	0.178024688	1	19	RNF103
primary alcohol metabolic process (GO:0034308)	**0.046466455**	0.178024688	1	19	ADH4
regulation of signal transduction by p53 class mediator (GO:1901796)	**0.046684796**	0.178024688	2	138	EXO1;ATR
cellular response to DNA damage stimulus (GO:0006974)	**0.049035416**	0.184528015	3	329	ETAA1;EXO1;ATR
**GO_Cellular_Component_Enrichment**					

Term	P-value	Adjusted P-value	Genes from top 50 list	Total genes	Genes
nuclear replication fork (GO:0043596)	0.027165345	0.298372914	1	11	ETAA1
NuRD complex (GO:0016581)	0.039272921	0.298372914	1	16	APLP2
CHD-type complex (GO:0090545)	0.039272921	0.298372914	1	16	APLP2
platelet alpha granule membrane (GO:0031092)	0.041676655	0.298372914	1	17	APLP2
**GO_Molecular_Function_Enrichment**					
**Term**	**P-value**	**Adjusted P-value**	**Genes from top 50 list**	**Total Genes**	**Genes**
oxidoreductase activity, acting on NAD(P)H, quinone or similar compound as acceptor (GO:0016655)	0.000629902	0.05480148	2	15	ADH4;AKR1C1
oxidoreductase activity, acting on paired donors (GO:0016709)	0.004719495	0.09951002	2	41	AKR1C1;CYP4F12
small GTPase binding (GO:0031267)	0.010854501	0.09951002	2	63	AFDN;APLP2
alcohol dehydrogenase activity, zinc-dependent (GO:0004024)	0.014908285	0.09951002	1	6	ADH4
retinol binding (GO:0019841)	0.014908285	0.09951002	1	6	ADH4
ketosteroid monooxygenase activity (GO:0047086)	0.014908285	0.09951002	1	6	AKR1C1
aldehyde dehydrogenase [NAD(P)+] activity (GO:0004030)	0.014908285	0.09951002	1	6	ADH4
5'-3' exodeoxyribonuclease activity (GO:0035312)	0.014908285	0.09951002	1	6	EXO1
oxidoreductase activity, acting on paired donors, with incorporation or reduction of molecular oxygen (GO:0016713)	0.017371741	0.09951002	1	7	CYP4F12
insulin-like growth factor II binding (GO:0031995)	0.017371741	0.09951002	1	7	IGFBP1
flap endonuclease activity (GO:0048256)	0.017371741	0.09951002	1	7	EXO1
single-stranded DNA exodeoxyribonuclease activity (GO:0008297)	0.017371741	0.09951002	1	7	EXO1
hormone activity (GO:0005179)	0.018324143	0.09951002	2	83	RETNLB;GRP
mismatch repair complex binding (GO:0032404)	0.019829161	0.09951002	1	8	ATR
bile acid binding (GO:0032052)	0.019829161	0.09951002	1	8	AKR1C1
alcohol dehydrogenase (NAD) activity (GO:0004022)	0.019829161	0.09951002	1	8	ADH4
oxidoreductase activity, acting on the CH-OH group of donors, NAD or NADP as acceptor (GO:0016616)	0.020450528	0.09951002	2	88	ADH4;AKR1C1
histone demethylase activity (H3-K36 specific) (GO:0051864)	0.022280559	0.09951002	1	9	KDM4C
exodeoxyribonuclease activity, producing 5'-phosphomonoesters (GO:0016895)	0.024725949	0.09951002	1	10	EXO1

lipid phosphatase activity (GO:0042577)	0.027165345	0.09951002	1	11	PLPPR5
phosphatase activity (GO:0016791)	0.027901009	0.09951002	2	104	PPP4R1;PLPPR5
alditol:NADP+ 1-oxidoreductase activity (GO:0004032)	0.029598762	0.09951002	1	12	AKR1C1
histone demethylase activity (H3-K9 specific) (GO:0032454)	0.032026213	0.09951002	1	13	KDM4C
water transmembrane transporter activity (GO:0005372)	0.032026213	0.09951002	1	13	AQP4
water channel activity (GO:0015250)	0.032026213	0.09951002	1	13	AQP4
phosphatidate phosphatase activity (GO:0008195)	0.032026213	0.09951002	1	13	PLPPR5
ubiquitin protein ligase activity involved in ERAD pathway (GO:1904264)	0.032026213	0.09951002	1	13	RNF103
insulin-like growth factor I binding (GO:0031994)	0.032026213	0.09951002	1	13	IGFBP1
insulin-like growth factor binding (GO:0005520)	0.034447714	0.103343143	1	14	IGFBP1
5'-3' exonuclease activity (GO:0008409)	0.036863279	0.106643205	1	15	EXO1
oxidoreductase activity, acting on the CH-CH group of donors, NAD or NADP as acceptor (GO:0016628)	0.041676655	0.106643205	1	17	AKR1C1
arachidonic acid epoxygenase activity (GO:0008392)	0.041676655	0.106643205	1	17	CYP4F12
arachidonic acid monooxygenase activity (GO:0008391)	0.041676655	0.106643205	1	17	CYP4F12
retinol dehydrogenase activity (GO:0004745)	0.041676655	0.106643205	1	17	ADH4
alcohol dehydrogenase (NADP+) activity (GO:0008106)	0.04885255	0.118033817	1	20	AKR1C1
aldo-keto reductase (NADP) activity (GO:0004033)	0.04885255	0.118033817	1	20	AKR1C1

Supplementary Table S3: KEGG pathway enrichment from the 1349 genes based on the expression in brain related tissues associated in the GWAS analysis**.**

Term	p-value	Adjusted p-value	Genes from 1349 list	Total Genes	Genes
Systemic lupus erythematosus	8.071E-12	2.36479E-09	34	133	*HIST1H2BM;C1S;HIST1H2BJ;HIST1H2BI;HIST2H4B;C7; HIST1H3F;HIST1H2AD; HIST1H3G;HIST1H2AG;HIST1H3H; HLA-DOA;HIST1H3B;HIST1H3D;HIST1H2AB;HIST1H3E; HIST1H2AI;HIST1H2AH; ACTN1;HIST1H2AJ;ACTN4;HIST1H4A; HIST2H3A;HIST1H4B;HIST4H4; HIST1H2BF;HIST1H2BE; HIST2H3D;HIST1H4D;HIST1H2BD;HIST1H4E;HLA-DRB1; HIST1H2BC;HIST1H4F*
Focal adhesion	2.6659E-11	3.9055E-09	42	199	*ITGB1;GSK3B;LAMA5;TNXB;FLT1;LAMA2;SHC1;ITGB3; PXN;LAMC2;LAMC1; THBS2;EGFR;THBS4;IGF1R;CCND1; AKT2;ERBB2;KDR; CAPN2;FLNB;FLNC;PDGFRB;PDGFRA; VWF; LAMB2;ACTN1;LAMB4;FN1;ACTN4;IGF1;PARVB;VEGFA; COL1A1;MYLPF; COL4A2;COL4A1;MYL2;COL6A2;COL4A3; COL6A3;ITGA7*
ECM-receptor interaction	4.6932E-10	4.58374E-08	24	82	*ITGB1;LAMA5;TNXB;VWF;LAMA2;LAMB2;ITGB3; LAMB4;FN1;LAMC2;LAMC1; THBS2;HSPG2;THBS4; COL1A1;COL4A2;COL4A1; COL6A2;COL4A3;DAG1;ITGA7; COL6A3;CD36;AGRN*
Human papillomavirus infection	3.167E-09	2.31985E-07	53	330	*ITGB1;GSK3B;PATJ;IFNA7;ITGB3;LAMC2;CHD4;LAMC1; RBPJ;IFNA8;CCND1; AKT2;CREB3L2;PDGFRB;VWF;TSC2; TNFRSF1A;CCNE2; COL4A2;COL4A1; COL6A2;COL4A3; COL6A3;ITGA7;LLGL2;NOTCH3;LAMA5;TNXB;LAMA2; NOTCH4;PXN;THBS2; PRKCZ;EGFR;THBS4;PPP2CB;HES1; TCF7L1; FZD2;JAG1;FZD5;FZD4;LAMB2;LAMB4;FN1; PPP2R3A;VEGFA;COL1A1;DLG3; APC;GNAS;KRAS;ATR*
Alcoholism	1.1422E-08	6.69356E-07	35	180	*HIST1H2BM;DDC;HIST1H2BJ;SHC1;HIST1H2BI;CAMKK2; HIST2H4B; CREB3L2;HIST1H3F;HIST1H2AD;HIST1H3G; HIST1H2AG;HIST1H3H; HIST1H3B;DRD2;HIST1H3D;*

					HIST1H2AB; HIST1H3E;HIST1H2AI; HIST1H2AH;HIST1H2AJ; HIST1H4A;HIST2H3A;HIST1H4B; HIST4H4;HIST1H2BF; GNAS;HIST1H2BE;KRAS; HIST2H3D;HIST1H4D;HIST1H2BD; HIST1H4E; HIST1H2BC;HIST1H4F
Pathways in cancer	2.1908E-08	1.06982E-06	71	530	*ITGB1;GSK3B;IFNA7;IL5RA;LAMC2;LAMC1; BRCA2;IFNA8;GLI3;IGF1R;EDNRA; MECOM;CCND1;CDH1;AKT2;JAK3;PDGFRB; PDGFRA; ARHGEF12;APAF1;IL15; DAPK2;MMP2;IL13; TGFBR2;ADCY9;COL4A2;CCNE2;MSH3;COL4A1; KIT;COL4A3;PPARG;IL6ST; NOTCH3;LAMA5;CTBP2; LAMA2;CTBP1;EPAS1;NOTCH4;MGST3;LRP5; TGFA;STK4; EGFR;LRP6;GNA11; TPR;ERBB2;CTNNA1;HES1;RXRG; TCF7L1;FZD2;JAG1;SMAD3;FZD5; GADD45B;FZD4; LAMB2;LAMB4;FN1;IGF2;IGF1;EPOR;VEGFA;CXCL12; APC;GNAS;KRAS*
PI3K-Akt signaling pathway	9.2376E-08	3.86662E-06	52	354	*ITGB1;GSK3B;IFNA7;FLT1;IRS1;ITGB3;LAMC2; LAMC1; IFNA8;IGF1R;CCND1; AKT2;CREB3L2;KDR;JAK3;PDGFRB; PDGFRA;VWF;TSC2;CCNE2;COL4A2; COL4A1;COL6A2; KIT;COL4A3; COL6A3;ITGA7;LAMA5;TNXB;LAMA2;TGFA; FOXO3;THBS2;EGFR;THBS4; PPP2CB;ERBB2;PCK1;MCL1;ANGPT2;LAMB2;INSR; LAMB4;IGF2;FN1;IGF1; PPP2R3A;EPOR;VEGFA;COL1A1; PKN2;KRAS*
Cholesterol metabolism	5.9868E-07	2.19268E-05	15	50	*ABCA1;SCARB1;LRPAP1;LRP1;APOA2;APOC3;LRP2;NPC2; APOH;LIPG; CD36;LDLRAP1;LPA;APOB;LDLR*
Tight junction	9.7627E-06	0.000317831	28	170	*ITGB1;PATJ;PRKAG2;F11R;PRKCZ;EPB41L4B;PPP2CB; CCND1;ERBB2; MYH14;SYNPO;MPDZ;ACTN1;ACTN4; MYH2;DLG3; CLDN9;MYL2;CLDN7;RAB13;MYH8;RAPGEF2; NF2; EZR;MYH4;CFTR;LLGL2;TJP2*
AMPK signaling pathway	1.5463E-05	0.000453074	22	120	*IRS1;INSR;ADIPOQ;TSC2;PRKAG2;IGF1;PPP2R3A;FOXO3;CPT1B; ACACB; IGF1R;CAMKK2;PPP2CB;CCND1;AKT2;HNF4A;CREB3L2; PPARG;CD36;PCK1; PPARGC1A;CFTR*
PPAR signaling pathway	2.7079E-05	0.000721279	16	74	*GK;ADIPOQ;APOA2;UCP1;NR1H3;APOC3;CPT1B;SCP2; ACOX2;EHHADH; PPARG;ACSBG1;CD36;ACAA1;PCK1;RXRG*

Amoebiasis	6.5888E-05	0.001608753	18	96	*LAMA5;RAB5C;SERPINB13;LAMA2;IL1R1;LAMB2;ACTN1; LAMB4;FN1;LAMC2;LAMC1;ACTN4;COL1A1; COL4A2;COL4A1; GNA11;COL4A3; GNAS*
Adherens junction	7.5485E-05	0.00170131	15	72	*TCF7L1;SMAD3;ACTN1;INSR;PTPRM;ACTN4;BAIAP2; PTPRF;EGFR;IGF1R;TGFBR2;CDH1;ERBB2;CTNNA1;NECTIN3*
Viral carcinogenesis	8.5655E-05	0.00179263	29	201	*HIST1H2BM;SP100;HIST1H2BJ;HIST1H2BI;HPN;PXN; CHD4;RBPJ;HIST2H4B; CCND1;CREB3L2;JAK3;GSN;ACTN1; ACTN4;HIST1H4A; HIST1H4B;CCNE2; HIST4H4;HIST1H2BF; HIST1H2BE;KRAS;LTBR;IL6ST;HIST1H4D;HIST1H4E; HIST1H2BD; HIST1H4F; HIST1H2BC*
Insulin resistance	0.0001026 9	0.001881295	19	108	*MLXIP;GSK3B;IRS1;INSR;PRKAG2;NR1H3;PYGM;CPT1B; PRKCZ;ACACB;PTPRF; TNFRSF1A;TBC1D4;AKT2;CREB3L2;CD36;SLC27A3;PCK1; PPARGC1A*
Thyroid hormone synthesis	0.0001049 4	0.001881295	15	74	*HSPA5;GPX3;ITPR1;GPX7;ITPR3;LRP2;TTF1;ATP1B1; ADCY9;TG;CREB3L2;CANX;GNAS;SLC26A4;DUOX2*
Dilated cardiomyopath y (DCM)	0.0001091 5	0.001881295	17	91	*ITGB1;RYR2;LAMA2;ITGB3;TNNC1;TPM1;IGF1;TTN; ADCY9;MYL2;TNNT2;LMNA;DAG1;GNAS;ITGA7; CACNA1S;MYH6*
Breast cancer	0.0001320 4	0.002149267	23	147	*NOTCH3;GSK3B;TCF7L1;FZD2;JAG1;FZD5; GADD45B;SHC1;FZD4;NOTCH4;LRP5; IGF1;BRCA2;EGFR;IGF1R;LRP6;CCND1;APC; AKT2;KIT; ERBB2;HES1;KRAS*
Small cell lung cancer	0.0001441 9	0.002223522	17	93	*ITGB1;LAMA5;APAF1;LAMA2;GADD45B;LAMB2;LAMB4; FN1;LAMC2;LAMC1;CCND1;CCNE2;COL4A2; COL4A1; AKT2;COL4A3;RXRG*
Hypertrophic cardiomyopath y (HCM)	0.0001570 2	0.002300345	16	85	*ITGB1;RYR2;LAMA2;ITGB3;TNNC1;TPM1;PRKAG2;IGF1; TTN;MYL2;TNNT2;LMNA;DAG1;ITGA7;CACNA1S;MYH6*

MAPK signaling pathway	0.00019985	0.002788423	37	295	*FLT1;TGFA;CACNA1A;STK4;EGFR;IGF1R;STK3;PPP3CC; MECOM;AKT2;ERBB2;KDR; FLNB;FLNC;CACNA1S;MAP3K6;MAP2K5;DUSP4;PDGFRB;PDGFRA; ANGPT2;GADD45B;IL1R1;INSR;PLA2G4B;IGF2; NFATC3;IGF1;VEGFA;TNFRSF1A; TGFBR2;JMJD7-PLA2G4B;KIT;TAOK2;NF1;RAPGEF2;KRAS*
Protein digestion and absorption	0.00031166	0.004150776	16	90	*COL18A1;COL14A1;ELN;KCNJ13;PRCP;ATP1B1;COL1A1; SLC7A8;COL4A2;COL5A1;COL4A1;COL6A2; COL5A3;COL4A3;COL5A2;COL6A3*
Endometrial cancer	0.00041372	0.005270461	12	58	*GSK3B;TCF7L1;CCND1;GADD45B;APC;CDH1;AKT2;ERBB2; CTNNA1;KRAS;FOXO3;EGFR*
Hippo signaling pathway	0.00046752	0.005524396	23	160	*GSK3B;PATJ;TCF7L1;SMAD3;FZD2;FZD5;FZD4;WWC1; BMP8A; PRKCZ;STK3;BMP5; TGFBR2;PPP2CB;CCND1;DLG3; APC;CDH1;CTNNA1; FAT4;NF2;LLGL2;TEAD3*
Proteoglycans in cancer	0.00047741	0.005524396	27	201	*ITGB1;CD63;ITGB3;PXN;ITPR1;ITPR3;EGFR; IGF1R; CCND1;AKT2;ERBB2;KDR; FLNB;FLNC;FZD2;ARHGEF12; FZD5;FZD4; MMP2; IGF2;FN1;ANK3;IGF1;HSPG2; VEGFA;KRAS;EZR*
Mineral absorption	0.00049022	0.005524396	11	51	*MT2A;TF;VDR;MT1M;MT1G;CYBRD1;MT1X;MT1H; ATP1B1;SLC39A4;FTL*
Adipocytokine signaling pathway	0.00062715	0.006805764	13	69	*IRS1;ADIPOQ;PRKAG2;CPT1B;ACACB;TNFRSF1A; CAMKK2;AKT2;ACSBG1;CD36; PCK1;RXRG;PPARGC1A*
Prostate cancer	0.0007383	0.007601937	16	97	*PDGFRB;PDGFRA;GSK3B;TCF7L1;TGFA;IGF1;EGFR; IGF1R;ZEB1;CCND1;SPINT1;CCNE2;AKT2;CREB3L2; ERBB2;KRAS*
Transcriptional misregulation in cancer	0.00075241	0.007601937	25	186	*FLT1;MLLT3;AFF1;IGF1R;HHEX;HIST1H3F;HIST1H3G; HIST1H3H; HIST1H3B;RXRG;HIST1H3D;SSX1;HIST1H3E; TAF15;GADD45B; PBX3;IGF1;TGFBR2;MAF;HIST2H3A;ZEB1; SPINT1;BMP2K;PPARG;HIST2H3D*
Insulin signaling pathway	0.00087481	0.008543939	20	137	*GSK3B;IRS1;SHC1;INSR;TSC2;PRKAG2;PYGM;PHKA2;PRKCZ; ACACB;PTPRF;HK1;SOCS1;PRKAR1B;AKT2; PHKG2;FLOT1;KRAS;PCK1;PPARGC1A*

Gastric cancer	0.00104697	0.009895575	21	149	*GSK3B;TCF7L1;SMAD3;FZD2;FZD5;GADD45B;SHC1;FZD4;LRP5;EGFR;TGFBR2;LRP6;CCND1;CCNE2;APC;CDH1;AKT2;ERBB2;CTNNA1;KRAS;RXRG*

Supplementary Material 1: Migraine data collection sheet

Patient ID:

Age:

Marital status

Menstrual history: Menarche: Duration of the period: regularity:

Use of oral contraceptives: Yes No

Migraine history:

Onset of the disease:

Type of migraine:

1. With aura
2. Without aura

Frequency of the headache:

Number of attacks per month:

1. 1
2. 2
3. 4
4. 8

5. 16
6. 28

Duration of the attack:

1. 4 hours
2. 8hours
3. 12 hours
4. 24 hours
5. 48 hours
6. 72 hours
7. >72 hours

Severity of the attack:

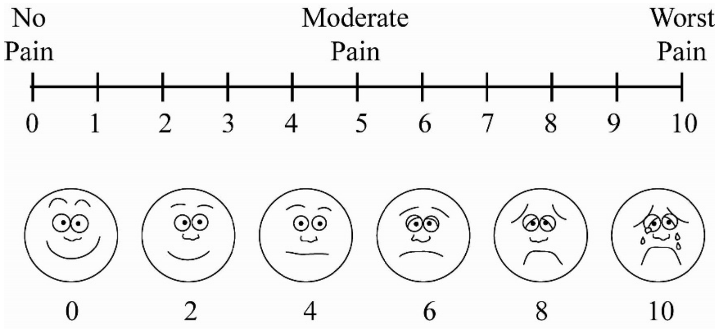

Use of medications:

1. No medication
2. Pain killer only
3. Prophylactic treatment only
4. Both pain killer and prophylactic.

Associated symptoms:

1. Nausea
2. Vomiting
3. Visual (blurring of vision, tunnel vision, streaks, flash light)
4. Auditory
5. Cutaneous.

Triggering factors:

1. Stress
2. Sleep disturbances
3. Weather changes
4. Skipped meal
5. Certain type of food
6. Coffee
7. Bright lights
8. Excessive noise
9. Strong smell
10. Physical exertion
